# Greater Trochanteric Fixation Using a Cable System for Partial Hip Arthroplasty: A Clinical and Finite Element Analysis

**DOI:** 10.1155/2014/931537

**Published:** 2014-08-10

**Authors:** Fırat Ozan, Şemmi Koyuncu, Mahmut Pekedis, Taşkın Altay, Hasan Yıldız, Gökhan Toker

**Affiliations:** ^1^Department of Orthopedics and Traumatology, Kayseri Training and Research Hospital, 38010 Kayseri, Turkey; ^2^Department of Orthopedics and Traumatology, İzmir Bozyaka Training and Research Hospital, 35110 İzmir, Turkey; ^3^Department of Mechanical Engineering, Faculty of Engineering, Ege University, 35040 İzmir, Turkey

## Abstract

The aim of the study was to investigate the efficacy of greater trochanteric fixation using a multifilament cable to ensure abductor lever arm continuity in patients with a proximal femoral fracture undergoing partial hip arthroplasty. Mean age of the patients (12 men, 20 women) was 84.12 years. Mean follow-up was 13.06 months. Fixation of the dislocated greater trochanter with or without a cable following load application was assessed by finite element analysis (FEA). Radiological evaluation was based on the distance between the fracture and the union site. Harris hip score was used to evaluate final results: outcomes were excellent in 7 patients (21.8%), good in 17 patients (53.1%), average in 5 patients (15.6%), and poor in 1 patient (9.3%). Mean abduction angle was 20.21°. Union was achieved in 14 patients (43.7%), fibrous union in 12 (37.5%), and no union in 6 (18.7%). FEA showed that the maximum total displacement of the greater trochanter decreased when the fractured bone was fixed with a cable. As the force applied to the cable increased, the displacement of the fractured trochanter decreased. This technique ensures continuity of the abductor lever arm in patients with a proximal femoral fracture who are undergoing partial hip arthroplasty surgery.

## 1. Introduction

The majority of intertrochanteric fractures in the elderly result from a fall while standing or walking [1]. The risk of falls increases with advancing age owing to systemic diseases, decreased physical capacity, and mobility impairment while standing or walking. This situation leads to an increased incidence of multiple comminuted and unstable fractures in elderly patients with osteoporosis [1, 2]. It is of utmost importance that the continuity of the abductor lever arm is ensured during partial arthroplasty for such fractures, including trochanteric fractures [3–6].

As the degree of displacement increases, nonunion presenting with reduced hip abductor muscle functions may occur for trochanteric fractures involving the abductor lever arm [3]. Impaired function or dysfunction of the abductor lever arm may result in pain, tenderness, an increased risk of hip dislocation, a Trendelenburg gait pattern, and reduced quality of life [4–7].

Various techniques have been developed—from using cerclage wire to specific trochanteric plate systems—to reduce the incidence of trochanteric nonunion and to ensure continuity of the abductor lever arm [3–13]. Cables are often used to increase stabilization of fractures and osteotomies [14]. Constant tension of the cables allows permanent compression of the bone fragments and fracture healing with reduced mobility [14].

In this study, the efficacy of greater trochanteric fixation using a multifilament cable to ensure continuity of the abductor lever arm in patients with a proximal femoral fracture undergoing partial hip arthroplasty was investigated.

## 2. Materials and Methods

Between 2009 and 2012, a total of 332 patients underwent hemiarthroplasty. Thirty-two of 98 patients (12 males and 20 females) who underwent greater trochanteric fixation with a cable were included. The mean age of the patients was 84.12 years (range 80–91 years). The mean follow-up was 13.06 months (range 6–29 months). A fall caused the fracture in all patients. Altogether, 19 patients (59.3%) had a right hip fracture, and 13 (40.7%) had a left hip fracture. According to the Evans-Jensen classification system [15, 16], 23 patients (71.8%) had type III trochanteric fractures, and 9 (28.1%) had type V trochanteric fractures. The mean duration of surgery was 70 min (range 60–90 min).

### 2.1. Surgical Technique

**Figure 1:**
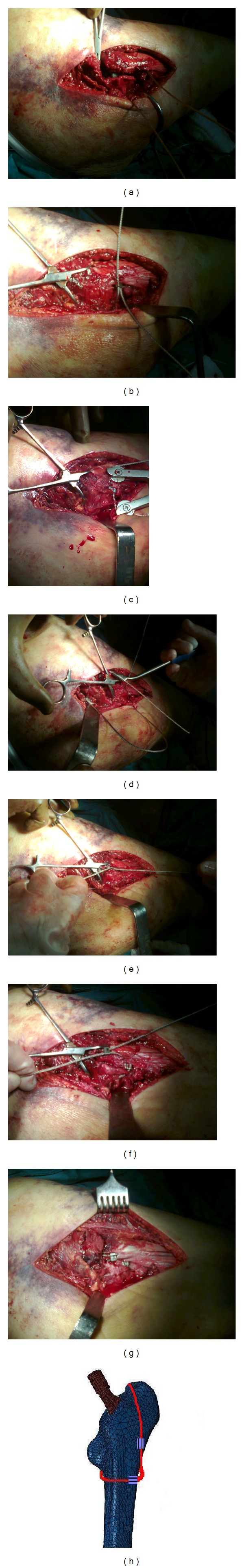
((a), (b), and (c)) Image of a greater trochanteric fracture including the abductor lever arm and the initial fixation at the level of the lesser trochanter. ((d) and (e)) The longer tip of the cable was advanced by a cable passer below the abductor muscle group and above the greater trochanter. ((e), (f), (g), and (h)) Greater trochanteric fracture was left in the reduction position and stretched using the shorter tip of the cable and a cable tensioner. The locking device was then squeezed.

**Figure 2:**
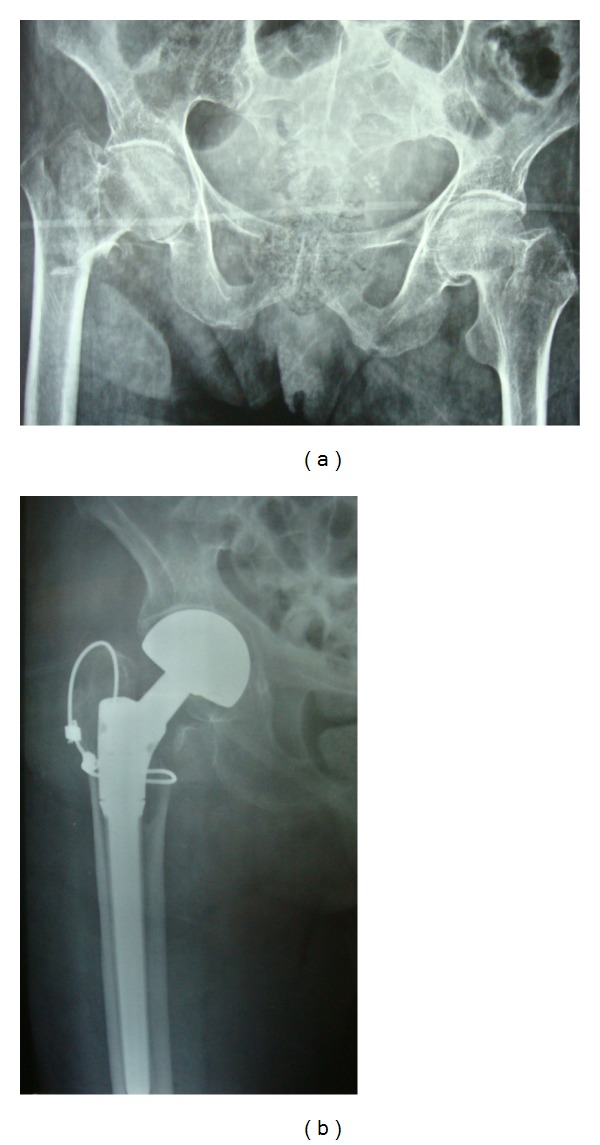
Radiographic images of a hip fracture. (a) Before surgery. (b) At 6 months after fixation with a cable.

All patients were administered thromboembolic prophylaxis with low molecular weight heparin 12 hours prior to surgery. Treatment continued for 10 days with a daily dose. First generation cephalosporin (cefazolin) 1 g in combination with prophylactic antibiotherapy was administered 30 min before surgery. In the postoperative period, gentamicin sulphate 160 mg was added for five days. The patients were placed in a lateral recumbent position. A modified Hardinge incision was made to reach the skin, subcutaneous layer, and fascia lata. Gluteus medius and vastus lateralis were reached. The authors advanced through the trochanteric fracture line and its damaged muscular region to reach the joint capsule. The femoral head was removed. Surgery was undertaken using the conventional bipolar hip arthroplasty technique. The cable (Zimmer, Warsaw, IN, USA) was advanced through the trochanter level and fixed to the femur by a cable passer to surround the muscular tissue following hip reduction (Figure 1(a)). One of the cable tips, which was left longer, was stretched by a cable tensioner and fixed to the femur (Figures 1(b) and 1(c)). A cable passer was then used to advance this longer tip of the cable below the abductor muscle group and above the greater trochanter (Figures 1(d) and 1(e)). The greater trochanteric fracture was left in the reduction position and immobilized. It was stretched using the shorter tip of the cable and a cable tensioner at a mean of 300 N (range 200–400 N). The locking device was then squeezed (Figures 1(f), 1(g) and 1(h)). Hip motion was checked, and the surgical zone was closed (Figures 2(a) and 2(b)). Surgery is usually performed by a specialist and a minimum of two residents in the authors' clinic. Four surgeons, that is, an assistant professor and three specialists, were involved in performing the current surgery.

The patients were mobilized with free loading using a four-leg walking assistance at an extent which they could tolerate within a week to prevent long-term postoperative immobilization-related complications. The patients discharged within 10 days after surgery based on their health status were scheduled for a visit at one month and three months and biannually thereafter.

### 2.2. Finite Element Analysis

Computed tomography images of 1.5 mm thickness of a healthy human left femur (male, 48 years) were obtained. Next, the surfaces of this model were constructed from these images using MIMICS 13.0 software (Materialise Inc., Leuven, Belgium). Note that reconstruction of this femoral model is based on a stack of CT slices. Following selection of an appropriate threshold value for the region of interest, the femoral bone was separated from the soft tissue. After the bone was labeled correctly in all slices of the CT scans, an automatic three-dimensional (3D) reconstruction of the hard tissue was obtained, creating triangulated surfaces. It should be noted that, for clear formation of 3D femoral bone from the slices, it is important to remove all of the holes and small islands in the labels. Next, the position of the model and its stem was adjusted to apply appropriate boundary conditions. Finally, numerical analysis was performed after importing the endoprosthetic femoral model into the ABAQUS 6.11 (Dassault Systèmes Simulia Inc., Providence, RI, USA) to simulate various cases using the explicit FEA module.

The present FE model was not representative of the patient population in terms of age and diagnosis. However, it was used because it represented an appropriate trochanteric image in this trochanteric fracture model.

**Figure 3:**
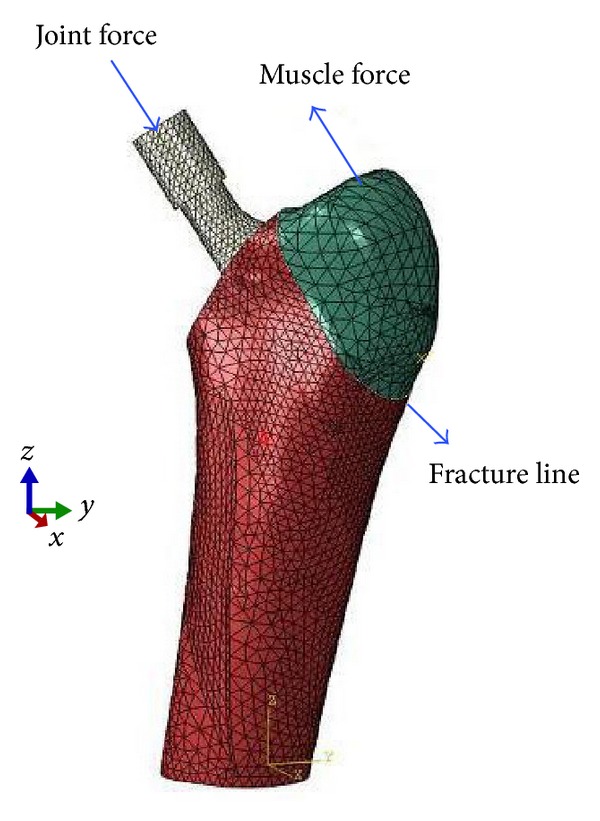
FEA model of the femoral bone and loading conditions.

Poisson's ratio of the femoral bone was 0.3, the elastic modulus was 17 GPa, and the density was 1590 kg/m^3^. Conversely, Poisson's ratio of the stem was 0.33, the elastic modulus was 113.8 GPa, and the density was 4428 kg/m^3^ [17]. The femoral bone and stem were modeled by deformable C3D10 M solid elements having 10 nodes, whereas the cable was modeled with a linear, rigid CONN3D2 connector-type element having two nodes that could transfer the loads axially. The femoral model and stem were modeled with 73.873 and 22.276 elements, respectively. The friction coefficient at the trochanteric fracture surface was selected as 1.0 (Figure 3) [18]. This value was chosen to decrease the large translational and rotational movement of the fractured trochanter.

The stem was mounted consistent with the surgical modality, and its position was adjusted based on the coordinate system of Bergmann et al. [19]. A 70° fracture line was produced in the trochanter. Loadings were performed on the *x*, *y*, and *z* coordinate system in the greater trochanteric site corresponding to the stem and abductor lever (Table 1) [20]. The distal part of the femur was fixed on three planes to prevent translation and rotation of the femur. After the preprocessing was completed, the next step was to perform a series of nonlinear finite analyses for two cases (walking, climbing) by applying varying loading values (200, 300, 400, and 500 N) to the cables to assess the performance of the multifilament cable fixation technique in terms of total displacement of the fractured greater trochanter.

### 2.3. Radiological Examination

Healing was defined as the presence of continuity between the greater trochanteric fracture and the adhesion site, as shown by anteroposterior radiography of the hip. Fibrous union was defined as a distance of ≤1.5 cm and nonunion as >1.5 cm between the adhesion site and the trochanteric fragment after its proximal migration [7]. All patients were assessed according to the Harris hip score (HHS) system during the final checkup [21].

### 2.4. Statistical Analysis

Statistical analysis was performed using SPSS version 16.0 software (SPSS Inc., Chicago, IL, USA). Qualitative variables were analyzed using the *χ* ^2^ test. A value of *P* < 0.05 was considered statistically significant.

## 3. Results

**Figure 4:**
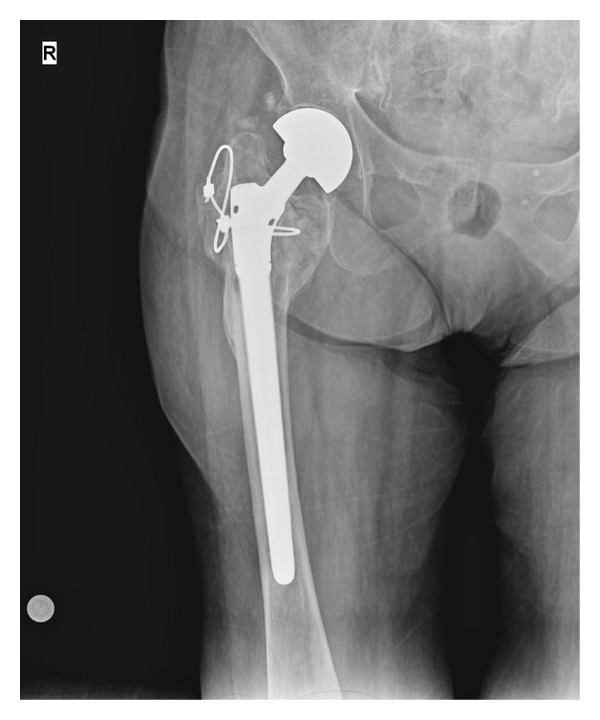
Image of a greater trochanteric fracture union.

**Figure 5:**
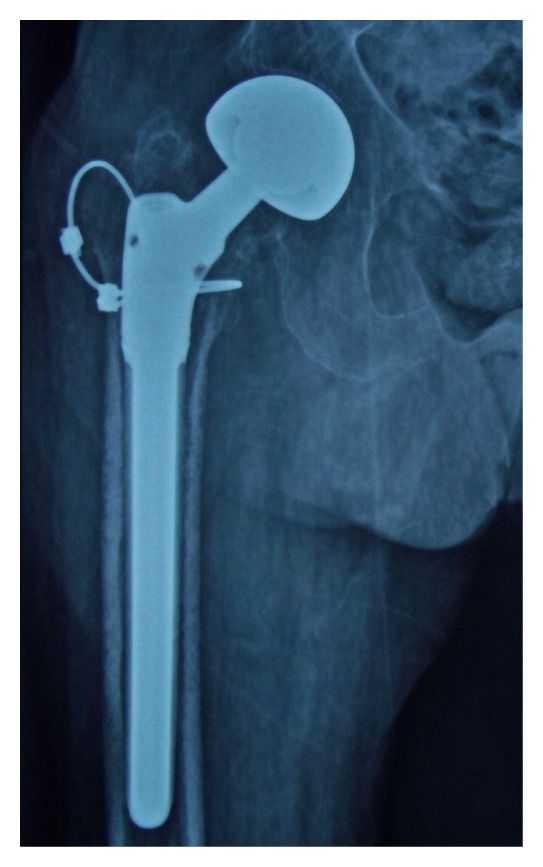
Image of fibrous union of a greater trochanteric fracture.

The mean HHS was 62.93 (range 32–78). According to the HHS, seven patients (21.8%) had an excellent outcome, 17 patients (53.1%) a good outcome, 5 patients (15.6%) an average outcome, and 1 patient (9.3%) a poor outcome. The mean abduction angle was 20.21° (range 5–35°). Union of the greater trochanteric fracture was achieved in 14 patients (43.7%) (Figure 4), fibrous union in 12 patients (37.5%) (Figure 5), and no union in 6 patients (18.7%). Eight patients (25.0%) had a positive Trendelenburg's sign, one (3.1%) had a broken cable, and one (3.1%) had trochanteric bursitis and was treated conservatively.

There was a significant relation between the HHS and union of the trochanteric fracture (*χ* ^2^ = 19.689; df = 6; *P* = 0.003). Excellent and good outcomes were significantly associated with a negative Trendelenburg sign (*χ* ^2^ = 9.503; df = 1; *P* = 0.002). Union of the greater trochanter was significantly associated with a negative Trendelenburg sign (*χ* ^2^ = 17.205; df = 2; *P* = 0.000).

The mean duration of surgery was 70 min (range, 60 to 90 min). Hemiarthroplasty with trochanteric fracture fixation prolonged the duration of surgery about 15 min in patients with proximal femoral fractures.

**Figure 6:**
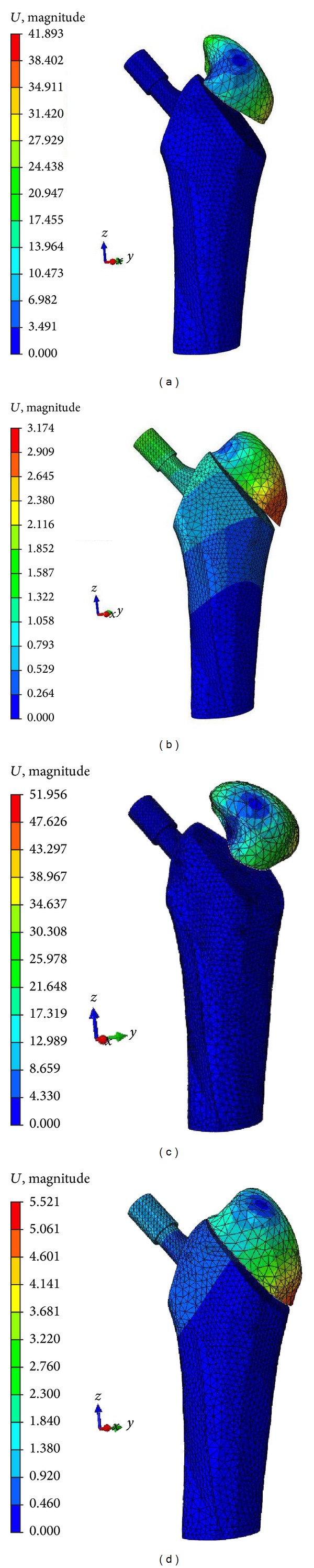
FEA results. Displacement of a greater trochanteric fracture without (a) and with (b) fixation by a cable, in the walking condition. Displacement of a greater trochanteric fracture without (c) and with (d) fixation by a cable, in the climbing condition. Cable tension was 300 N. Displacement was measured in millimeters.

Displacements of the greater trochanteric fracture with or without fixation using a cable during walking and climbing, on the *x*, *y*, and *z* dimensions, are shown in Figure 6. Numerical results showed that the maximum displacement areas were mainly located in the bottom regions of the trochanter (where muscle force is applied) and with less intensity in the distal bone. Higher maximum displacements were found in patients who sustained their fractures while climbing than in those who were walking. More force applied to the cable resulted in less displacement of the trochanteric fracture (Table 2).

## 4. Discussion

This study showed that greater trochanteric fracture fixation using a cable to maintain the continuity of the abductor lever arm in hemiarthroplasty is an easy method with some equipment with prolonged duration of surgery (about 15 min). Based on the FEA, prevention the proximal femoral bone migration of the greater trochanteric fracture, maintained a relative stability. Due to the short duration of follow-up in the current study, it can be concluded that the symptoms of patients with fibrous union and trochanter major nonunion, in particular, are suggestive of delayed union due to the short duration of follow-up. Therefore, small sample size and short follow-up are the main limitations to this study. In addition, early mobilization with loading to the hip to prevent postoperative immobilization-related complications might worsen the healing process of relatively stable trochanteric fracture.

Abductor muscle forces of the hip carry the body weight, producing vertical, anteroposterior, and rotational forces on the greater trochanter. For satisfactory fixation, the trochanteric site must exert resistance to these forces by compressing the cancellous surfaces [22]. Vertical forces alone result in at least a twofold increase in the body's weight during walking, and anteroposterior shear forces produce nearly a fourfold increase in body weight during climbing or standing up [10]. Contractions of the gluteus medius and minimus produce a rotational force on the trochanter through short external rotators [11]. In addition, there is a significant imbalance in terms of strength between trochanteric migration distally via the vastus lateralis and proximally via abductor muscles [22].

Unfixed trochanteric fractures may cause impaired function of the abductor muscles of the hip following partial hip arthroplasty, with the most common complication being trochanteric nonunion. Impaired function or dysfunction of the abductor lever arm may result in pain, increased risk of hip dislocation, and a Trendelenburg gait pattern [3–6, 9, 12].

Various techniques, including reducing trochanteric nonunion by fixation, have been described to ensure the continuity of the abductor lever arm [9, 13]. However, there is, as yet, no consensus on the best approach for the cable grip system [9]. The surgery is lengthy and there is substantial blood loss in patients undergoing surgery for a trochanteric fracture [3]. Fixation of a greater trochanteric fracture can be achieved using a standard wire or cable grip system. In the literature, the rate of nonunion for greater trochanteric fractures was reported to be 37.5% when using a standard wire or cable grip system [4]. The nonunion rate in the study described here was 18.7%. In another study, the union rate of greater trochanteric fractures was 91% in 223 patients who underwent total hip arthroplasty using the Dall-Miles cable grip system [23].

Clinical failure in the literature is defined as ≥1 cm of proximal migration of the greater trochanteric fracture [3, 7, 9, 13]. Amstutz and Maki [24] showed that abductor muscle weakness was directly associated with the migration rate of the greater trochanteric fracture. Abductor muscle weakness was clinically confirmed in patients with a 2 cm migration of the greater trochanteric fracture. A 1 to 2 cm migration of the greater trochanteric fracture was used as an endpoint for evaluating the deformity [3, 7, 9, 13]. Healing was defined as the presence of continuity between the greater trochanteric fracture and the adhesion site, as shown by radiography of the hip. Fibrous union was defined as a distance of ≤1.5 cm and nonunion as >1.5 cm between the adhesion site and the trochanteric fragment after its proximal migration.

In a study that included 56 patients undergoing acetabular cup revision, Panousis et al. [13] performed trochanteric fixation through a Chevron osteotomy with a cerclage wire. The authors reported a fibrous union rate of 7.1%, a nonunion rate of 5.3%, and a ruptured wire rate of 17.8%. The current study reports a union rate of 37.5% and a nonunion rate of 18.7%.

Loosening of the cables can occur perioperatively or postoperatively. It is often caused by soft tissue interposition and micromovement and migration secondary to bone resorption [14]. The cable failure rate is between 27% and 44% [14]. The present authors observed fixation failure in two patients (6.2%), including one with a ruptured cable and one with a loosened cable.

The difference in the migration rate was estimated to be 92.4% for fixed hips with a cable versus an unfixed hip model in the walking condition. In contrast, with the FEA in the climbing condition, the difference was 89.4% after fixation with a cable (cable tension of 300 N). Additionally, as more force was applied to the cable, there was less displacement of the trochanteric fracture. Therefore, it can be suggested that greater trochanteric fixation using a cable enables adequate stability for fracture union by preventing proximal displacement and maintaining sufficient compression on the cancellous surfaces.

In this study, a hip model with FEA to evaluate the efficacy of trochanteric fixation with a cable was created. A 70° fracture line was produced in the trochanter to reduce fracture stability and it was an unstable fracture model. On the other hand, FEA analysis has several limitations. First, a wide range of fracture patterns in real-life setting can be observed. The geometry created using FEA was not patient-specific. In addition, the hip is exposed to repetitive loads while walking or climbing in the real-life setting. However, in this study, in order to reduce the CPU (Central Processing Unit) solution time, the loads that acted on human hip for one step were evaluated during the analysis.

Second, osteoporosis may cause reduced bone density, potentially leading to an altered elastic modulus. However, the current FEA showed proximal femoral fracture migration with a rigid body motion over time. Therefore, it is believed that osteoporosis may have only slightly affected the elastic modulus, but this would need to be investigated more thoroughly in the future.

Third, applying an extensive force to the wires can crush the trochanter in osteoporotic bones. When bone is osteoporotic, applying too large of a force to the wires can crush the trochanter. However, it is unknown if the 200 to 500 N forces currently applied to the cable to estimate the total amount of the bone migration would cause permanent damage to the trochanter. Only the linearity of force-migration was analyzed.

Finally, 3D cortical bone exhibits orthotropic behavior with three orthogonal planes. However, a limitation of this study is that the cortical bone was modeled as a linear isotropic material. It is well established that the efficiency and convenience of numerical methods depend highly on a number of factors including accuracy of the associated geometrical models, suitability and accuracy of the material models, and knowledge of the nature of the phenomenon and the boundary conditions.

## 5. Conclusion

Based on the mean age of the patients and the duration of surgery, it can be suggested that greater trochanteric fixation through a multifilament cable is a simple, although time-consuming, method that ensures continuity of the abductor lever arm for partial hip arthroplasty.

## Figures and Tables

**Table 1:** Femoral component and abductor lever arm load on *x*, *y*, and *z* planes in the walking and climbing conditions via finite element analysis.

Site	Walking			Climbing		
	*x*	*y*	*z*	*x*	*y*	*z*
Hip contact	451.44∗	−451.44	−1916.112	502.271	−513.282	−2001.46
Abductor	−540.892	127.072	674.652	−703.01	301.532	654.731

Source: Heller et al. [20].

∗All results (loads) are given in newtons (N).

**Table 2:** Maximum displacement rates of great trochanteric fractures after loading on *x*, *y*, and *z* planes via finite element analysis.

	Trochanteric fracture without cable fixation				Trochanteric fracture with cable fixation				Cable tension (N)
	*u*_*x*_	*u*_*y*_	*u*_*z*_	*u*_total_	*u*_*x*_	*u*_*y*_	*u*_*z*_	*u*_total_	
Walking (mm)	5.36	36.70	50.67	62.73	0.87	4.13	2.38	4.69	200
	3.64	24.53	33.75	41.89	0.51	2.71	1.55	3.17	300
	2.73	18.50	25.21	31.11	0.42	2.12	1.15	2.31	400
	2.28	14.68	20.35	25.23	0.34	1.53	0.82	1.80	500

Climbing (mm)	10.27	45.29	62.78	77.23	2.01	5.46	5.65	8.38	200
	6.78	30.39	41.58	51.95	1.34	3.73	3.84	5.52	300
	5.01	22.69	31.79	38.56	1.19	2.68	2.76	4.24	400
	4.37	18.23	24.25	31.57	0.70	2.04	2.29	3.41	500
